# Failure of Alzheimer’s Mice Brain Resident Neural Precursor Cells in Supporting Microglia-Mediated Amyloid β Clearance

**DOI:** 10.3390/cells11050876

**Published:** 2022-03-03

**Authors:** Marva Lachish, Nina Fainstein, Tal Ganz, Lihi Sofer, Tamir Ben-Hur

**Affiliations:** 1 Department of Neurology, The Agnes Ginges Center for Human Neurogenetics, Hadassah-Hebrew University Medical Center, Jerusalem 91120, Israel; marva.lachish@mail.huji.ac.il (M.L.); nina.fainstein@mail.huji.ac.il (N.F.); talganzlg@gmail.com (T.G.); lihi.sofer@mail.huji.ac.il (L.S.); 2 Faculty of Medicine, Hebrew University of Jerusalem, Jerusalem 91120, Israel

**Keywords:** Alzheimer’s disease, microglia, neural precursor cells, amyloid β, phagocytosis, degradation

## Abstract

The failure of brain microglia to clear excess amyloid β (Aβ) is considered a leading cause of the progression of Alzheimer’s disease pathology. Resident brain neural precursor cells (NPCs) possess immune-modulatory and neuro-protective properties, which are thought to maintain brain homeostasis. We have recently showed that resident mouse brain NPCs exhibit an acquired decline in their trophic properties in the Alzheimer’s disease brain environment. Therefore, we hypothesized that functional NPCs may support microglial phagocytic activity, and that NPCs derived from the adult AD mouse brain may fail to support the clearance of Aβ by microglia. We first identified in the AD brain, in vivo and ex vivo, a subpopulation of microglia that express high Aβ phagocytic activity. Time-lapse microscopy showed that co-culturing newborn NPCs with microglia induced a significant increase in the fraction of microglia with high Aβ phagocytic activity. Freshly isolated NPCs from adult wild type, but not AD, mouse brain, induced an increase in the fraction of microglia with high Aβ phagocytic activity. Finally, we showed that NPCs also possess the ability to promote Aβ degradation within the microglia with high Aβ phagocytic activity. Thus, resident brain NPCs support microglial function to clear Aβ, but NPCs derived from the AD environment fail to do so. We suggest that the failure of AD brain NPCs to support Aβ clearance from the brain by microglia may accelerate disease pathology.

## 1. Introduction

Both the familial and late-onset forms of Alzheimer’s disease (AD) manifest with early deposition of amyloid β (Aβ) in the brain, occurring decades before clinical presentation of dementia symptoms [1,2]. Deposition of Aβ induces a cascade of pathologic changes that create a toxic brain environment, which promotes neurodegeneration. Aβ also stimulates a state of chronic neuroinflammation, manifesting with marked astro- and micro-gliosis [3].

Microglia, the brain innate immune cells, are increasingly appreciated as key players in the pathogenesis of AD and other neurodegenerative diseases [4,5]. It is thought that early in the course of disease they function to remove excess Aβ from accumulating in the brain, but eventually fail in this task [6]. Microglia are activated to a neurotoxic state by misfolded Aβ and phosphorylated tau [4,7]. This state of massive activation of microglia also displays paradoxical functional failure, contributing to the progression of AD by failing to clear Aβ plaques, and damaging neurons via nitric oxide, reactive oxygen species, and cytokine production [8].

Neural precursor cells (NPCs) possess powerful immune-modulatory, neurotrophic, and neuroprotective properties [9,10]. NPC transplantation into the CNS has shown beneficial effects in multiple preclinical models of various diseases [11,12]. Specifically, the transplantation of NPCs in AD models attenuated neuroinflammation by inhibition of microgliosis, reduction of pro-inflammatory cytokine expression [13,14], improved Aβ deposition/clearance ratio [15], improved mitochondrial function [16], enhanced endogenous neurogenesis [17], and improved memory impairment, in association with induction of BDNF-enhanced synaptogenesis [18]. The apparent therapeutic properties of transplanted NPCs underscore the question whether these represent the main physiological role of resident NPCs in the brain throughout life. Furthermore, these properties suggest that resident brain NPCs may have a role in protecting the brain from degeneration, and that their functional failure may be associated with disease progression. Indeed, we recently showed that resident brain NPCs from a transgenic model of AD exhibit defective immune-modulatory and neurotrophic properties, as compared to wild type NPCs [19]. Importantly, this failure was not inherent, but acquired during the accumulation of brain pathology.

Previous studies have suggested that among their tissue-supporting functions, NPCs affect microglial functions. Specifically, NPCs increase microglial proliferation, chemotaxis, activation state and phagocytic activity [20]. In addition, NPCs reverse activated microglia into an anti-inflammatory phenotype. This process is mediated by secreted factors such as TGFβ [21], PGE2 [22], and secreted exosomes [23], as well as by forming direct connections through gap junctions [24]. Microglia modulation by NPCs may have protective effects on neuronal survival, as has been shown in co-culture systems with organotypic brain slices. Therefore, we hypothesized that brain pathology in AD may induce functional failure of resident NPCs in supporting microglia. In particular, we hypothesized that AD brain-derived NPCs may fail to support microglial ability to engulf and process Aβ, leading to exacerbation of disease.

To test this, we examined the trophic effect of primary NPCs (isolated from wild type versus 5XFAD murine brains) on the phagocytic functions of freshly isolated microglia from brains of the transgenic murine 5xFAD model in a co-culture system ex vivo. We describe a subpopulation of microglia in the AD brain which displays high-Aβ phagocytic activity. We show that NPCs from both newborn and adult WT brains induce an increase in this microglial population. We show that NPCs derived from adult mouse AD brains fail to support microglial phagocytic activity.

## 2. Materials and Methods

### 2.1. Animal Models

A colony of 5xFAD mice was initially established by mating C57Bl/6 J mice (originally supplied by Jackson, Bar Harbor, ME, USA) with heterozygote 5xFAD mice (5xFAD transgenic mouse model, carrying five mutated human genes associated with familial Alzheimer’s disease, APP K670N/M671L (Swedish), APP I716V (Florida), APP V717I (London), PS1 M146L, PS1 L286V); both were generous gifts from Professor Dani Frenkel of Tel Aviv University. A colony of 5xFAD-nestin-GFP was established by mating heterozygote 5xFAD mice with C57Bl/6 mice expressing green fluorescent protein (GFP) under the nestin promoter, which were generous gifts from Professor Eli Keshet of the Hebrew University, with the permission of Gregory Enikilopov from Cold Spring Harbor, New York. 

Offspring of further generations were screened for carrying the transgenic cassettes and/or expressing GFP (see below). To ascertain that 5xFAD and wild type (WT) control mice had identical genetic backgrounds and environments, further breeding was performed by mating a heterozygote transgene-positive with a transgene-negative mouse, and all littermates were housed together. In the 5xFAD-nestin-GFP colony, the same method was performed with one specification, i.e., that all mice were GFP-positive. We made sure that each litter contained approximately 50% transgene-positive mice. Both male and female mice were used, maintaining equal distribution between experimental groups. Newborn mice were obtained from the C57BL/6JOLAHSD colony (Envigo, Ness Ziona, Israel). Animal experimentation was approved by the institutional ethics committee, approval number MD-18-15671-3.

### 2.2. Mice Genetic Screening

Mice tails were sampled for polymerase chain reaction (PCR) analysis of APP, PS1, GFP, and a control mouse gene. DNA was extracted from mice tails using 50 mM Tris HCl (pH = 8), 0.05% Triton X-100, and 19.6 mg/mL Proteinase K. The vials were then heated to 55 °C for 15 min followed by 5 min in 80 °C. The PCR reaction mixture included 5 μL of DNA, 300 nM of the appropriate forward and reverse primers (Syntezza, Jerusalem, Israel), and 5 μL of a master mix buffer containing nucleotides and Red Load Taq polymerase (Larova, Jena, Germany) in a total volume of 25 μL. Gene amplification was carried out using the GeneAmp 9700 Sequence Detection System (Applied Biosystems, Bedford, MA, USA). For APP analysis, amplification included one stage of 3 min at 94 °C, followed by 35 cycles of a three-step loop: 30 s at 94 °C, 1 min at 55 °C, and 1 min at 72 °C, followed by 2 min in 72 °C and cooling to 10 °C. For PS1 analysis, amplification included 35 cycles of a three-step loop: 20 s at 94 °C, 1 min at 60 °C, and 1 min at 72 °C, followed by 2 min in 72 °C and cooling to 10 °C. For GFP analysis, amplification included one stage of 10 min at 94 °C, followed by 25 cycles of a three-step loop: 30 s at 94 °C, 30 s at 55 °C, and 30 s at 72 °C, followed by 5 min in 72 °C and cooling to 20 °C.

### 2.3. Isolation and Growth of Microglia

Microglia were isolated from 7-month-old 5xFAD mice. Mice were terminally anesthetized and decapitated, and their brains were excised into Earle’s based salt solution. The extraction was performed in a sterile environment. Brain tissue was dissociated to a single-cell suspension using a neural tissue dissociation kit (P) (130-092-628, Miltenyi Biotec, Bergisch Gladbach, Germany), according to the manufacturer’s protocol, followed by Myelin removal using Percoll (GE17-0891-01, Sigma-Aldrich-Merck, St. Louis, MO, USA). Sorting for CD11b+ cells was achieved by magnetic CD11b (Microglia) MicroBeads (130-093-634, Miltenyi Biotec), according to the manufacturer’s protocol followed by depletion with MS columns (130-042-201, Miltenyi Biotec), resulting in CD11b+ single-cell suspension. Cells were counted and plated on Poly-D-Lysine (10 µg/1 mL, Sigma-Aldrich-Merck) coating of either 24-well plate cover slips (150,000 per well), 35 mm plate (Ibidi, Gräfelfing, Germany, 81158, 850,000 cells, for a time-lapse experiment, described below) or 6-well plate cover slips (1 million per well, for RNA extraction, described below). Cells were grown in a microglia medium (20% FBS, 30% L-cells conditioned medium, 1% Pen-strep, 1% L-glutamine, 1% sodium Pyruvate, DMEM low glucose), for 3 days prior to activation. For activation, LPS (0.2 µg/1 mL Sigma-Aldrich-Merck, L2630) was added for 24 h.

### 2.4. Isolation and Growth of Adult Mouse SVZ-Derived NPCs

Subventricular zone (SVZ)-derived NPC isolation was performed on 7-month-old 5xFAD and WT mice. The mice were terminally anesthetized and decapitated, and their brains were excised into Earle’s based salt solution. Each brain was put into a brain slicer and three 1 mm thick slices between Bregma AP +1.5 and −1.5 were obtained. SVZ extracts were dissected from the slices using a fine tip glass pipette. The SVZ extracts from each mouse group were pooled and dissociated to a single-cell suspension using a neural tissue dissociation kit (P) (Miltenyi Biotec, 130-092-628) according to the manufacturer’s protocol. For myelin removal, the cells were incubated with Myelin Removal Beads II (Miltenyi Biotec, 130-096-733) according to the manufacturer’s protocol, followed by depletion with LS columns (Miltenyi Biotec, 130-042-401), resulting in a myelin-free single-cell suspension. The cells were counted and plated in a 24-well plate (300,000 cells per well) in a B27 (Gibco, Waltham, MA, USA, 12587010) supplemented DMEM/F12-GlutaMAX (Gibco) [19] medium. Growth factors EGF (R&D Systems, Minneapolis, MN, USA, 236-EG-01M, 0.02 µg/1 mL), FGF (R&D Systems,233-FB-025, 0.01 µg/1 mL) were added to the plate.

### 2.5. Isolation and Expansion of Nestin-GFP SVZ-NPCs

SVZ-derived NPC isolation was performed on 7-month-old 5xFAD nestin-GFP and WT nestin-GFP mice, as described previously. After receiving a myelin-free single-cell suspension, the GFP+ cells were sorted using BD FACS Aria III (BD Bioscience, Franklin Lakes, NJ, USA). The GFP+ SVZ-NPCs were plated (70,000–90,000 cells per well in a 96-well plate) in NPC medium. Growth factors EGF (0.02 µg/1 mL) and FGF (0.01 µg/1 mL) were added to the plate.

### 2.6. Isolation and Growth of Newborn Mouse NPCs

The animals were anesthetized on ice for 10 min and decapitated, and their brains were extracted into Earle’s based salt solution. Then, the brain stems were removed, followed by removal of the meninges under microscope, using forceps. Tissue dissociation was achieved by Trypsin (Sigma-Aldrich-Merck, T-4566, 5 mg/20 mL), and afterwards by the addition of Trypsin inhibitor (T-6522, 12.5 mg/1 mL, Sigma-Aldrich-Merck), DNAse (150,000 units, D-2025, Sigma-Aldrich-Merck) and magnesium sulfate (0.335 g/10 mL, Sigma-Aldrich-Merck). Collecting the cell suspension resulted in single NPCs. The cells were counted and plated (1 million cells/1 mL) in NPC medium (described above). Growth factors EGF (0.02 µg/1 mL) and FGF (0.01 µg/1 mL) were added to the flask.

### 2.7. Co-Culturing Microglia with NPCs

Adjacent to LPS activation, the NPCs were counted and added to microglial cultures (at a 1:2 microglia:NPC ratio for 5xFAD SVZ and newborn NPCs, and a 1.6:1 microglia:NPC ratio for sorted GFP+ NPCs) for 24 h. In order that the adult NPCs’ ex vivo cultures would represent their in vivo functions, NPC isolation occurred 2 days prior to co-culturing. For co-culturing with newborn NPCs, the NPCs were isolated one day prior to co-culturing, in order to resemble the sphere size of adult NPCs. For degradation experiments, the NPCs were co-cultured with microglia, after Aβ administration for 2 h, for 48 h (24 h after LPS activation). For RNA extraction, NPCs were co-cultured with microglia in a 6-transwell plate (8 µm polycarbonate membrane, Corning, Corning, NY, USA ) for 24 h following LPS activation.

### 2.8. Time-Lapse

Co-culturing for 24 h was followed by live cells staining with wheat germ agglutinin (WGA, W11263, Invitrogen™, Waltham, MA, USA) according to the manufacturer’s protocol. Then, the cells were imaged in a confocal microscope in incubator conditions (37 °C, 5% CO_2_) every 45 s for 30 min or 2 h upon addition of a fluorescent substance.

### 2.9. Phagocytosis and Degradation Assays

Phagocytosis was measured using two fluorescent substances. Hilyte-555 Aβ_1-42_ (2 µM, AnaSpec, Fremont, CA, USA) was added to the culture for 30 min or 2 h. Latex beads (L3030, Sigma-Aldrich-Merck) were added to the culture for 30 min. Degradation was measured using non-fluorescent Aβ_1-42_ (2 µM, A9810, Sigma-Aldrich-Merck) for 2 h, then the cells were washed with PBS followed by microglia medium replacement containing LPS for 48 h. At the end of the time course, staining was performed as previously described [25]. The following antibodies were used: rat anti-Trem2 (1:1000, R&D Systems), rat anti-MHC II (1:200, Santa Cruz, Dallas, TX, USA), rabbit anti-Iba1 (1:220, 019-19741, Wako, Richmond, VA, USA), mouse anti-Aβ (1:750, Biolegend, San Diego, CA, USA, SIG-39300). Goat anti-rat Alexa Fluor 488 (1:200, Invitrogen), goat anti-rabbit Alexa Fluor 488 (1:200, Invitrogen), and goat anti-mouse Alexa Fluor 555 (1:200, Invitrogen) were used as secondary antibodies where appropriate.

### 2.10. Histopathology

The animals were anesthetized with a lethal dose of pentobarbital and their brains were perfused via the ascending aorta with an ice-cold phosphate buffered saline, followed by cold 4% paraformaldehyde. The tissues were deep-frozen in dry ice, serial 10 µm coronal sections were prepared, and immunofluorescent stainings were performed as previously described [19,26]. Rabbit anti-Iba1 and mouse anti-Aβ were used for immune-fluorescent staining, as described above.

### 2.11. RNA Extraction and Real Time PCR

Total RNA was isolated from a 6-well plate of microglia using TRI Reagent (Molecular Research Center INC, Cincinnati, OH, USA), according to the manufacturer’s protocol [27]. cDNA was prepared from 100 ng of total RNA using a qScript cDNA synthesis kit (Quantabio, Beverly, MA, USA). For real time (RT) PCR, the reaction mixture included 1 mL of cDNA, 300 nmol/L concentrations of the appropriate forward and reverse primers (Syntezza, Israel), and 5 mL of SYBR green mix PerfeCTa SYBR Green FastMix Rox in a total volume of 10 mL. Gene amplification was carried out using the GeneAmp 7000 PCR system (Applied Biosystems). The gene expression results were normalized to the TBA gene. Five independent experiments were repeated in triplicate and presented as the SE of mean. For the mRNA quantification, the fold change was normalized to the TBA transcript (ΔCT).

### 2.12. Analysis

All images were quantified manually in Photoshop or ImageJ. Latex beads’ uptake was measured by the percentage of cells that uptake ≥3 latex beads per image frame. The high-Aβ phagocytic microglia subpopulation was separated by setting lower laser exposure and shorter exposure time duration in the confocal microscope. The microscope configuration was identical for each experiment and the fraction of Aβ+ cells was measured. The mean fluorescent intensity (MFI) was measured in the red/green channel using Photoshop for each specific manually defined cell frame. The cells were chosen randomly. Statistical analysis was performed with GraphPad Prism by using ANOVA, and post hoc multiple comparison analysis was performed by the Tukey test. In experiments comparing two groups, a one-sided Student *t*-test was used.

## 3. Results

### 3.1. Microglia from Adult AD Brain Exhibit an Increased Latex Beads’ Phagocytosis

**Figure 1:**
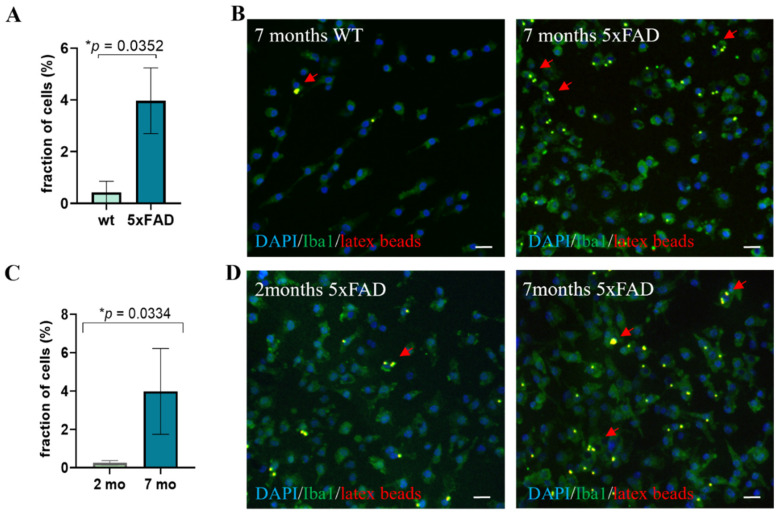
Characterization of latex beads uptake by microglia. (**A**) A higher fraction of microglia from 7-month-old 5xFAD mice exhibit high phagocytic activity (≥3) latex beads, as compared to microglia from 7-month-old wild type (WT) mice. Student *t*-test * *p* = 0.0352. (**B**) Representative images of latex beads’ uptake by microglia from 7-month-old WT and 5xFAD mice. (**C**) A higher fraction of microglia from 7-month-old 5xFAD mice exhibit high phagocytic activity of latex beads, as compared to 2-month-old 5xFAD mice. Student *t*-test *p = 0.0334. (**D**) Representative images of latex beads’ uptake by microglia from 2- and 7-month-old 5xFAD mice. Red arrows point at cells with ≥3 latex beads. Blue = DAPI, green = Iba1, red = latex beads, scale = 20 µM.

It is well known that the inflammatory AD brain environment is associated with activation of microglia [3]. To examine whether this affects the function of primary microglia following their isolation for ex vivo assays, we measured their phagocytic activity, as determined by latex beads’ uptake. In the 5xFAD model, at age 2 months there is almost no Aβ deposition and no microgliosis, whereas at age 7 months there is a marked increase of microglia numbers and markers of activation, as well as heavy deposition of Aβ [19,28]. Microglia were isolated from the brain by magnetic bead sorting of dissociated brain tissue for CD11b+ cells and cultured 4 days ex vivo. All adherent cells expressed Iba1. Microglia from 7-month-old 5xFAD mice exhibited a significant increase in phagocytosis of latex beads, as compared to microglia from WT mice (Figure 1A,B, *p* = 0.0352). This phenotype was acquired by the effect of the AD brain environment, as 7-month-old microglia from 5xFAD mice exhibited a significantly increased phagocytic activity compared to the microglia from 2-month-old 5xFAD mice (Figure 1C,D, *p* = 0.0334) [19,28,29]. Therefore, we performed further experiments on microglia from 7-month-old 5xFAD mice, showing increased latex beads’ phagocytic activity ex vivo.

### 3.2. A Subpopulation of Microglia in the AD Brain Exhibits High Aβ Phagocytic Activity

**Figure 2:**
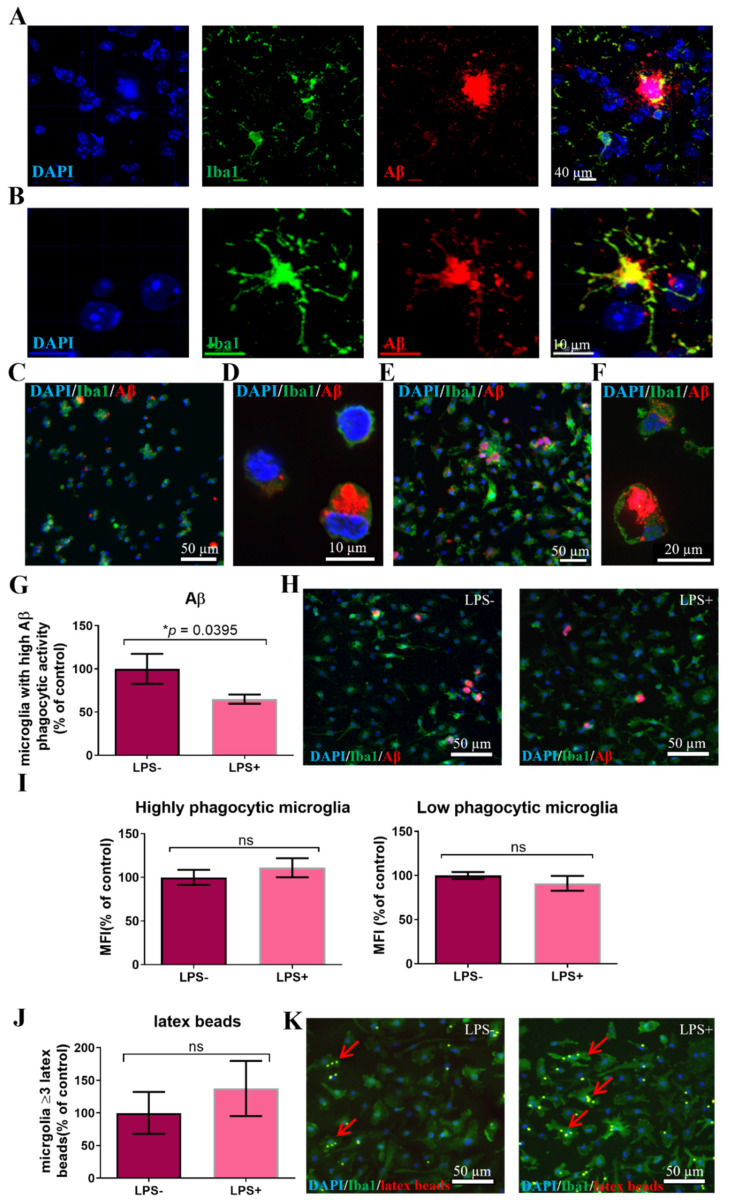
Characterization of Aβ uptake by microglia. (**A**) Immune-fluorescent staining of brain sections from 7-month-old 5XFAD mouse (Bregma AP = 0) for Aβ and Iba1+ cells show the presence of amyloid plaques surrounded by microglia. (**B**) High resolution microscopy of stained brain section shows microglia with massive intracellular Aβ accumulation. (**C**) Freshly isolated microglia from 7-month-old 5xFAD mice show varying amounts of intracellular Aβ. (**D**) A subpopulation of microglia from 7-month-old 5xFAD mice shows massive intracellular Aβ content. (**E**) Isolated microglia that were fed with fluorescent Aβ displayed varying levels of Aβ uptake; 5–10% of microglia exhibited high Aβ phagocytic activity. (**F**) Higher resolution shows microglia with low versus high Aβ phagocytic activity. (**G**) Incubation of microglia with LPS induced a decrease in the microglial subpopulation with high phagocytic activity of Aβ. Student *t*-test *p = 0.0395. (**H**) Representative images of microglial uptake of Aβ in the absence and presence of LPS. (**I**) Aβ amount per cell (by mean fluorescence intensity) did not change in response to LPS activation in either microglial populations with high or low Aβ phagocytic activity. (**J**) Incubation of microglia with LPS induced a non-significant increase in the fraction of microglia with ≥3 latex beads. (**K**) Representative images of microglial uptake of latex beads in the absence and presence of LPS. Blue = DAPI, green = Iba1, red = Aβ/latex beads, ns = non-significant, MFI = mean fluorescent intensity, scale = 50 µM.

Given the increase in latex beads’ uptake by adult 5XFAD brain-derived microglia, we next examined Aβ uptake by microglia. Extracellular deposition of Aβ occurs typically in plaques, surrounded by microglia [30]. The level of removal of extracellular Aβ by microglia is considered to be a major player in the brain’s ability to prevent neurodegeneration in AD and therefore, may be an important therapeutic target [31]. We first stained 7-month-old 5xFAD mouse brains, for Iba1+ microglia and Aβ, to examine the intracellular amyloid content within microglia. There were many extracellular Aβ+ plaques, surrounded by microglia (Figure 2A). Higher resolution showed many Iba1+ microglia with varying low amounts of cytoplasmic Aβ. There was a subpopulation of Iba1+ cells that displayed very strong intracellular Aβ staining (Figure 2B). A subpopulation of microglia with marked accumulation of intracellular Aβ was also observed in 5–10% of freshly isolated CD11b+ cells from 5xFAD brains, stained ex vivo for intracellular Aβ (Figure 2C,D).

Since the AD brain displayed a subpopulation of microglia with high Aβ content, we examined whether isolated microglia exhibit differential Aβ phagocytic activity that may indicate the existence of subpopulations with high- versus low-potential for Aβ removal. To that end, we incubated CD11b+ cells isolated from 7-month-old 5xFAD mice brains ex vivo with fluorescent-labeled Aβ peptide for 30 min. The majority of cells phagocytosed some Aβ, and a small subpopulation (5–10% of cells) showed marked Aβ uptake (Figure 2E,F). Then, to mimic the brain’s inflammatory environment in AD, we examined the effect of lipopolysaccharide (LPS) on Aβ uptake in general, and on the microglial subpopulation with high-phagocytic activity in particular. We used LPS for this purpose, given the vast literature on the role of endotoxins in AD and neurodegeneration [32]. LPS exacerbates brain pathology in animal models of AD and increases Aβ production and aggregation [33]. Clinical studies found significantly increased blood and brain endotoxin levels [34]. Endotoxin is specifically found in amyloid plaques, surrounded by microglia [35,36]. Therefore, CD11b+ microglia that were isolated from 7-month-old 5xFAD mouse brains were incubated for 3 days, then stimulated with LPS for 24 h prior to feeding with Aβ. LPS stimulation induced a significant decrease in the fraction of microglia with high phagocytic activity (Figure 2G,H, *p* = 0.0395), with no change in the mean fluorescent intensity (MFI) per cell of Aβ staining in both microglia populations exhibiting high and low Aβ phagocytic activity (Figure 2I). Aβ uptake by microglia occurs via receptor mediated phagocytosis. To assess whether LPS affects phagocytic activity in general, or receptor mediated specifically, we fed LPS-treated and untreated microglia with fluorescent latex beads for 30 min. LPS induced a trend of increase in phagocytosis of latex beads (Figure 2J,K).

Thus, the brain of AD mice contains a subpopulation of microglia with high Aβ phagocytic activity. LPS induces a mild increase in latex particle phagocytosis, but significantly reduces the fraction of microglia with high Aβ phagocytic activity. Therefore, the reduced Aβ uptake by cultured 5xFAD-derived microglia exposed to LPS may represent, in part, the failure in Aβ removal that occurs in the inflamed brain environment, inflicted with AD pathology. Accordingly, all additional experiments were performed on microglia that were isolated from 7-month-old 5xFAD mice and cultured ex vivo in the presence of LPS.

### 3.3. Neural Precursor Cells Induce an Increase in the Fraction of Microglia with High Aβ Phagocytic Activity

**Figure 3:**
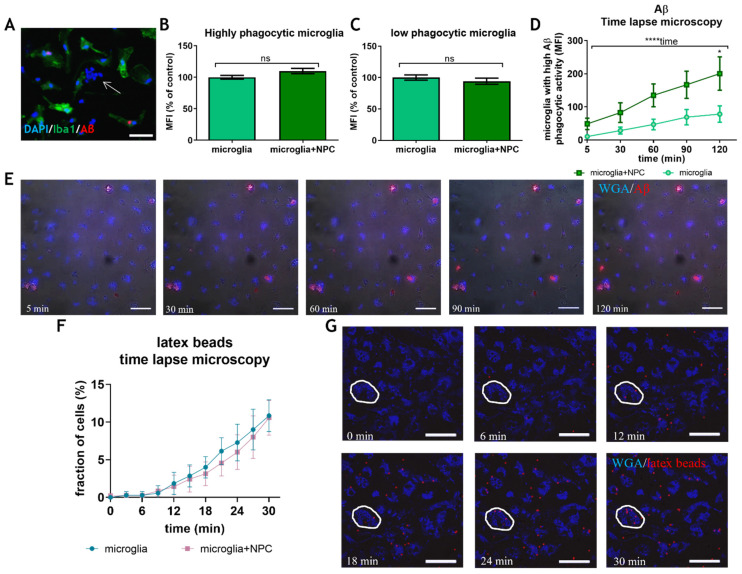
Time-lapse microscopy measuring the effect of newborn NPCs on microglial Aβ and latex beads’ uptake. (**A**) Fluorescent Aβ was found only in Iba1+ cells. Arrow = a cluster of NPCs. (**B**) Incubation of microglia with NPCs did not affect the amount of intracellular Aβ uptake at 2 h in microglia with high Aβ phagocytic activity. (**C**) Incubation of microglia with NPCs did not affect the amount of intracellular Aβ uptake at 2 h in microglia with low Aβ phagocytic activity. (**D**) Analysis of Aβ uptake by time-lapse microscopy was performed at five time points, showed that co-culturing with NPCs induced a significant two-fold increase in the fraction of microglia with high Aβ phagocytic activity. Two-way ANOVA and post hoc analysis showed a significant factor of time (**** *p* < 0.0001), and of NPC effect at 2 h (* *p* = 0.0184). (**E**) Representative images of time-lapse microscopy for Aβ uptake at the five time points. (**F**) Analysis of latex beads’ uptake by time-lapse microscopy was performed for 30 min and showed that co-culturing with NPCs did not affect the fraction of microglia with ≥3 latex beads’ uptake. (**G**) Representative images of time-lapse microscopy for latex beads’ uptake at six time points. The circle defines cell boundaries. Blue = WGA, red = Aβ/latex beads, ns = non-significant, MFI = mean fluorescent intensity, scale = 50 µM.

Neural precursor cells (NPCs) possess powerful trophic and immune-modulatory properties that have proved to be effective in protecting the brain in various disease models [9,37,38]. It has been proposed that their physiological role is to maintain brain homeostasis. We hypothesized that resident brain NPCs may regulate Aβ removal by microglia. Previous studies showed that NPCs induce an increase in latex beads’ phagocytosis by murine microglia [20], as well as by the human microglial cell line [39]. Here, we characterized NPCs’ effects on Aβ uptake by primary microglia, isolated from 7-month-old 5xFAD mice brains which we cultured short-term ex vivo in the presence of LPS. The 5xFAD brain-derived microglia were co-cultured with newborn brain NPCs for 24 h before feeding them with fluorescent Aβ. Upon Aβ administration, time-lapse microscopy was performed over the course of 2 h. Five time points were chosen for analysis from the starting point (approximately 5 min after Aβ administration) and every 30 min thereafter. We examined the effect of NPCs on the amount of intracellular Aβ in microglia with high and low Aβ phagocytic activity, and on the fraction of microglia with high Aβ phagocytic activity. First, we confirmed that Aβ uptake was by microglia rather than by the co-cultured NPCs by staining for Iba1 (Figure 3A). Co-culturing microglia with NPCs did not affect the amount of Aβ uptake in either microglial subpopulation with high and low Aβ phagocytic activity, as indicated by MFI measurements after 2 h (Figure 3B,C). However, NPCs induced a significant increase in the fraction of microglia with high Aβ phagocytic activity compared to control (Figure 3D,E, 2-way ANOVA, *p* = 0.018 for microglia + NPCs as compared to microglia alone at 2 h, *p* < 0.0001 for the effect of time on phagocytosis). Interestingly, high Aβ phagocytes accumulated throughout the 2-h experiment course, and NPCs induced an approximately two-fold increase in their number as compared to controls (Figure 3D). A difference in Aβ uptake was noticeable even at the starting point, probably since phagocytosis starts immediately, before time-lapse microscopy was initiated. Thus, NPCs do not significantly affect the amount of Aβ uptake per cell, but rather induce an increase in the fraction microglia with high Aβ phagocytic activity. We then examined whether NPCs also affect non-receptor mediated phagocytosis. Time-lapse microscopy of latex beads’ uptake by microglia did not identify any effect of NPCs, whether looking at the fraction of microglia that phagocytosed ≥3 latex beads (Figure 3F,G), or ≥7 beads (not shown). Thus, NPCs induce a selective effect on AD brain-derived microglia to increase the fraction of cells with high Aβ phagocytic activity.

**Figure 4:**
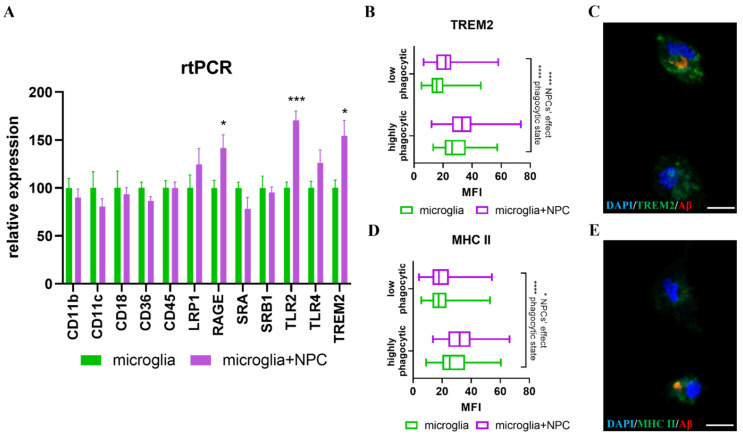
NPCs induce an increase in expression of genes associated with phagocytosis in microglia. (**A**) Real time PCR on RNA from microglia isolated from 7-month-old 5xFAD mice. Co-culturing with newborn-mouse-derived NPCs induced increased gene expression of RAGE (* *p* = 0.03), TLR2 (*** *p* = 0.0003) and TREM2 (* *p* = 0.016) in microglia. (**B**) Quantification of TREM2 staining by MFI per cell in high- and low-phagocytic microglia in the presence or absence of NPCs. Two-way ANOVA analysis showed significant factors of groups (1.2-fold increase in expression by NPCs, **** *p* < 0.0001) and phagocytic activity (1.5-fold higher expression in highly phagocytic microglia, **** *p* < 0.0001). (**C**) Representative image of TREM-2 expression in microglia with high versus low Aβ-phagocytic activity. (**D**) Quantification of MHC-II staining per cell by MFI in high- and low-phagocytic microglia in the presence or absence of NPCs. Two-way ANOVA analysis showed significant factors of groups (1.15-fold increase in expression by NPC in the high phagocytic group, * *p* < 0.0302) and phagocytic activity (1.46-fold expression in highly phagocytic microglia, **** *p* < 0.0001). (**E**) Representative image of MHC-II expression in microglia with high versus low Aβ phagocytic activity. There was no interaction between the two variables in B and D, suggesting that NPCs’ effect and the phagocytic state were independent in their effects on TREM2 and MHCII expression. MFI = mean fluorescent intensity. blue = DAPI, green = MHC-II/TREM2, red = Aβ, scale = 10 µM.

Since NPCs support receptor mediated phagocytosis, we examined the effect of NPCs on the expression of several membranal receptors and mediators of phagocytosis. NPCs induced a significant increase in the expression of TLR2, TREM-2 and RAGE mRNA in microglia (Figure 4A). To validate the RT-PCR results, we performed immune-fluorescent stainings for TREM-2 in microglia that were co-cultured with NPCs versus control microglia and fed them with Aβ in order to differentiate between cells with high versus low phagocytic activity. Incubation of microglia with NPCs induced a very mild but significant increase in TREM-2 protein expression in both microglia with high and low Aβ phagocytic activity. Importantly, the expression of TREM-2 was 1.5-fold higher in microglia with high Aβ phagocytic activity. Thus, higher TREM-2 expression characterizes highly phagocytic microglia, the fraction of which is increased by co-culturing with NPCs (Figure 4B,C). Also, recent studies indicated the presence of an activated microglial subpopulation in the AD brain, exhibiting high expression of MHC-II and high phagocytic activity [40]. We found that MHC-II expression was indeed higher in microglia with high Aβ phagocytic activity, and that co-culturing NPCs with microglia further increased MHC-II expression in this fraction of microglia (Figure 4D,E).

### 3.4. Wild Type NPCs Increase Aβ Phagocytosis by Microglia, but 5xFAD Subventricular Zone Extracts Enriched for NPCs Do Not

**Figure 5:**
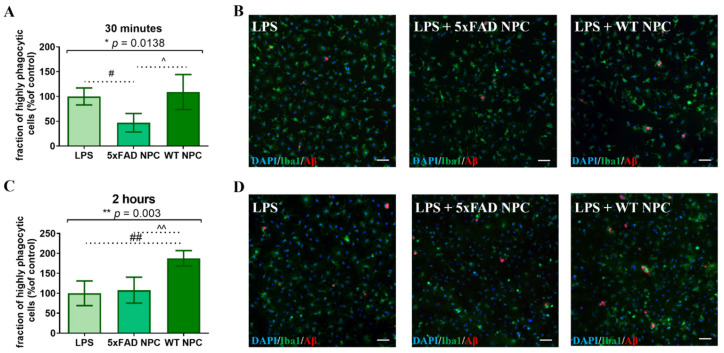
Dissociated SVZ explants (SVZ-NPC) from WT, but not 5xFAD mice, increase the fraction of microglia with high Aβ phagocytic activity. (**A**) Co-culturing of 5xFAD microglia with SVZ-NPC extracted from 5xFAD mice reduced the fraction of microglia with high Aβ phagocytic activity after 30 min. One-way ANOVA * *p* = 0.0138, post hoc analysis: 5xFAD SVZ-NPC vs. LPS # *p* = 0.0363, 5xFAD SVZ-NPC vs. WT SVZ-NPC ^ *p* = 0.0168. The fraction of microglia with high Aβ phagocytic activity in LPS (control condition) served as 100%. (**B**) Representative images of microglia with high Aβ phagocytic activity after 30 min (**C**) Co-culturing of 5xFAD microglia with SVZ-NPC from WT mice increased the fraction of microglia with high Aβ phagocytic activity after 2 h. One-way ANOVA: ** *p* = 0.003, post hoc analysis: WT NPCs vs. 5xFAD NPC ^^ *p* = 0.0078, WT NPCs vs. LPS ## *p* = 0.0044. The fraction of microglia with high Aβ phagocytic activity in LPS (control condition) served as 100%. (**D**) Representative images of microglia with high Aβ-phagocytic activity after 2 h. Blue = DAPI, green = Iba1, red = Aβ, scale = 50 µM.

Our previous work showed that the AD brain environment causes functional failure in the immune-modulatory and neurotrophic properties of resident brain NPC [19]. Therefore, we compared the ability of NPCs isolated from 7-month-old 5xFAD and wild type mice to support Aβ phagocytosis. Since NPCs’ propagation in vitro restores their functions [19], we used freshly isolated cells. First, we compared the effect of subventricular zone extracts that were enriched with NPCs (SVZ-NPC, containing approximately 5–7% nestin+ cells, not shown). Microglia were extracted from 7-month-old 5xFAD mice, incubated and stimulated with LPS for 24 h prior to Aβ administration. Crude SVZ explants were dissociated and co-cultured with microglia for 24 h, followed by the addition of fluorescent Aβ. Based on time-lapse microscopy findings on the dynamics of Aβ uptake, we measured the fraction of a microglial subpopulation with high Aβ phagocytic activity at 30 min and 2 h. At 30 min, the microglia co-cultured with 5xFAD SVZ-NPC exhibited a significant decrease in the fraction of microglia with high Aβ phagocytic activity, compared to control microglia and microglia co-cultured with wild type SVZ-NPC (Figure 5A,B, *p* = 0.0138 by ANOVA). At the 30 min time point, wild type SVZ-NPC had no significant effect on the fraction of microglia with high Aβ phagocytic activity as compared to control. An independent experiment at 2 h showed that wild type SVZ-NPC induced a significant increase in the fraction of microglia with high Aβ phagocytic activity, as compared to controls and to microglia co-cultured with 5xFAD SVZ-NPC (Figure 5C,D, *p* = 0.003 by ANOVA). 5xFAD SVZ-NPC had no effect on the microglia. Thus, SVZ-NPC derived from wild type mice induced an increase in the fraction of microglia with high Aβ phagocytic activity, while SVZ-NPC from 7-month-old 5xFAD mice failed to do so.

### 3.5. Isolated Nestin-GFP from Adult Wild Type NPCs, Induced an Increase in Microglial Aβ Phagocytosis, but Nestin-GFP 5xFAD NPCs Did Not

**Figure 6:**
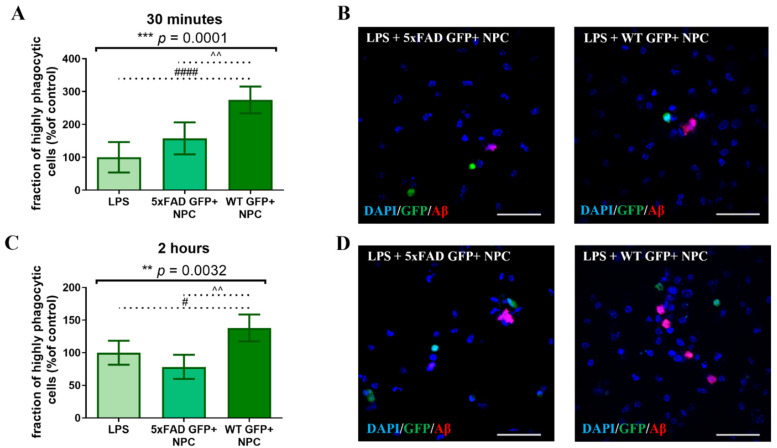
GFP+ NPCs extracted from SVZ of WT mice, but not 5xFAD mice, induced an increase in the fraction of microglia with high Aβ phagocytic activity. (**A**) Co-culturing of 5xFAD microglia with GFP+ NPCs extracted from WT mice showed an increased fraction of microglia with high Aβ phagocytic activity after 30 min. One-way ANOVA: *** *p* = 0.0001, post hoc analysis indicated that highly phagocytic microglia in the WT GFP+ NPCs group were increased compared to other conditions. WT GFP+ NPCs vs. 5xFAD GFP+ NPCs: ^^ *p* = 0.0081, WT GFP+ NPCs vs. LPS: #### *p* < 0.0001. The fraction of microglia with high Aβ phagocytic activity in LPS (control condition) served as 100%. (**B**) Representative images of microglia with high Aβ phagocytic activity after 30 min. (**C**) Co-culturing of 5xFAD microglia with GFP+ NPCs extracted from WT mice showed an increased fraction of microglia with high Aβ phagocytic activity after 2 h. One-way ANOVA: ** *p* = 0.0032, post hoc analysis indicated that highly phagocytic microglia in the WT GFP+ NPCs group were increased compared to other conditions. WT GFP+ NPCs vs. 5xFAD GFP+ NPCs: ^^ *p* = 0.0026, WT GFP+ NPCs vs. LPS: # *p* = 0.0256. The fraction of microglia with high Aβ phagocytic activity in LPS (control condition) served as 100%. (**D**) Representative images of microglia with high Aβ phagocytic activity after 2 h. Blue = DAPI, green = GFP, red = Aβ, scale = 50 µM.

Since SVZ-explants are enriched in NPCs, but comprised of other cell types as well, we further verified that their differential effect on Aβ uptake by microglia is mediated by NPCs. We crossed the 5xFAD mouse strain with a line expressing GFP under the nestin promoter [41]. Since nestin expression was not unique to NPCs and could be found also on blood vessel wall cells, we sorted by FACS the GFP+ cells (GFP-nestin cells) from adult mouse SVZs. The sorted cells generated NPC spheres, which could differentiate into neural progeny with high efficacy [19]. Wild type and 5xFAD GFP-nestin NPCs were co-cultured for 24 h with microglia that were isolated from 7-month-old 5xFAD mice and cultured in the presence of LPS prior to the addition of fluorescent Aβ for either 30 min or 2 h. Both independent experiments showed that at 30 min (Figure 6A,B, *p* = 0.0001 by ANOVA) and 2 h (Figure 6C,D, *p* = 0.0032 by ANOVA), wild type (but not 5xFAD) GFP-nestin NPCs induced an increase in the fraction of microglia with high Aβ phagocytic activity, as compared to control. Although the number of GFP-nestin NPC that were added to the co-cultures were approximately one-third of the dissociated SVZ-NPC, their effect was similar to that of SVZ-NPC. This was probably due to the higher fraction of functional NPCs in the FACS-sorted GFP-nestin cell population. Thus, freshly isolated wild type NPCs induced a significant increase in the fraction of microglia with high Aβ phagocytic activity, and NPCs isolated from 5xFAD brains failed to do so.

### 3.6. WT Newborn NPCs Increase Aβ Removal from 5xFAD Microglia

**Figure 7:**
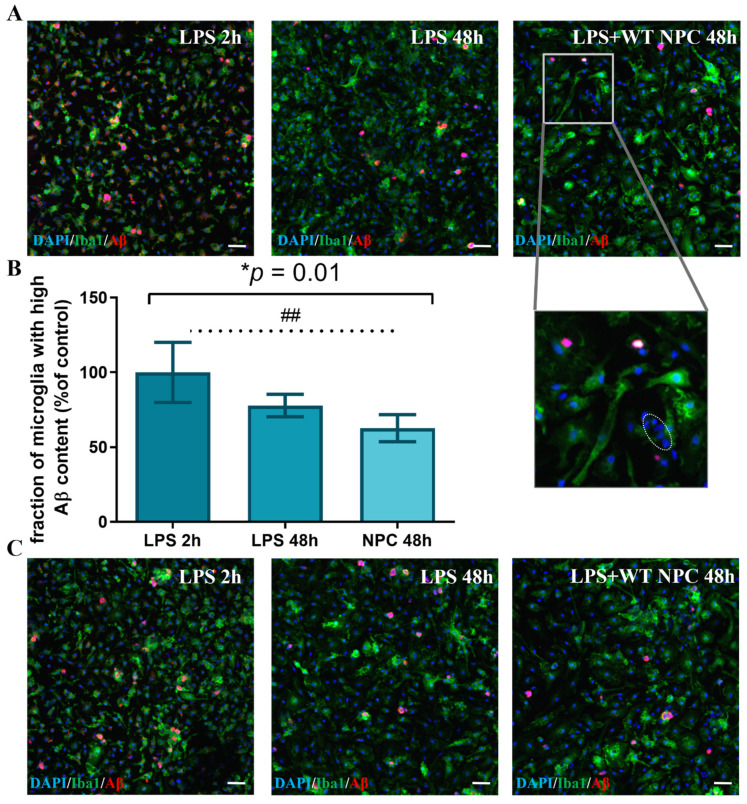
Newborn mouse NPCs promote Aβ removal in highly phagocytic microglia after 48 h. (**A**) After 48 h, almost no remaining Aβ was found in microglia with low Aβ phagocytic activity, with no difference between conditions. Representative images in high exposure show microglia with high and low Aβ content at the 2 h baseline, while only microglia with high Aβ content are observed after 48 h in control and NPC-treated cultures. Enlargement in the right panel shows a (circled) Iba1-negative cluster of NPCs. (**B**) Co-culturing Aβ-loaded 5xFAD microglia with NPCs from WT newborn mice induced a reduction of the fraction of microglia with high Aβ content after 48 h as compared to control. Baseline fraction of microglia with high Aβ content (LPS 2 h) served as 100%. One-way ANOVA: * *p* = 0.01, post hoc analysis indicated that NPCs promote degradation. NPC 48 h vs. LPS 2 h: ## *p* = 0.0087. (**C**) Representative images of microglia with high Aβ content at the 2 h baseline, and after 48 h in control and NPC-treated cultures. Blue = DAPI, green = Iba1, red = Aβ, scale = 50 µM.

The massive accumulation of Aβ in microglia drove us to examine its degradation, another critical element of the effective removal of excessive Aβ from the brain. The effect of NPCs on microglial degradation abilities is poorly studied. Microglia were extracted from 7-month-old 5xFAD mice and stimulated with LPS for 24 h. This was followed by an addition of Aβ for 2 h, before the free Aβ was washed out by replacing it with a fresh medium containing LPS. First, the effect of newborn WT mouse NPC spheres was examined by adding them to Aβ-loaded microglia for the rest of the culture period. Double immune-fluorescent staining was performed for Iba1 and Aβ. In microglia with low Aβ phagocytic activity, the Aβ content at 48 h was compared to the 2 h baseline (the timepoint in which the medium was replaced). At 48 h, almost no remaining Aβ was found in microglia with low Aβ phagocytic activity both in control cultures and those exposed to NPCs. (Figure 7A), suggesting complete removal. We then examined whether NPCs affect Aβ content in microglia with high phagocytic activity. There was a non-statistically significant trend of reduction in Aβ content per cell (data not shown). However, NPCs induced a significant reduction in the fraction of microglia with high Aβ content after 48 h. This suggests that NPCs support the clearance of Aβ in microglia with high Aβ content, although some microglia are resistant to NPCs’ effect and remain loaded with Aβ (Figure 7, * *p* = 0.01 by ANOVA). Post hoc analysis indicated that NPC spheres induce removal of Aβ by microglia (*p* = 0.0087), while control cultures showed a non-significant trend in the reduction in microglia with a high Aβ load. Thus, NPCs may affect both the uptake and the clearance of Aβ in microglia.

### 3.7. Nestin-GFP NPCs from Adult Mice Increases Aβ Removal from 5xFAD Microglia

**Figure 8:**
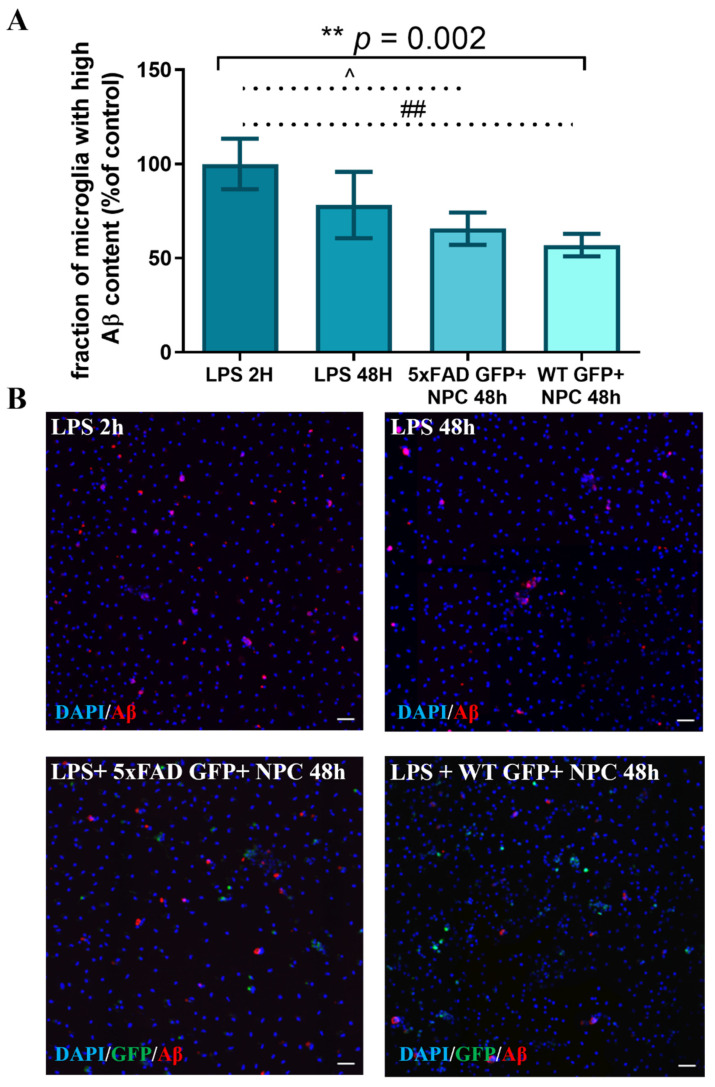
GFP+ NPCs from adult SVZ promote Aβ removal in microglia with high Aβ content after 48 h. (**A**) Co-culturing Aβ-loaded 5xFAD microglia with GFP+ NPCs induced a reduction of the fraction of microglia with high Aβ content after 48 h as compared to control. Quantification of microglia with high Aβ content was compared to control rate (LPS 2 h), which served as 100%. One-way ANOVA ** *p* = 0.002, post hoc analysis indicated that both WT and 5xFAD GFP+ NPCs promote Aβ removal. 5xFAD GFP+ NPCs 48 h vs. LPS 2 h: ^ *p* = 0.012, WT GFP+ NPCs 48 h vs. LPS 2 h: ## *p* = 0.0025. (**B**) Representative images of microglia with high Aβ content at the 2 h baseline, and after 48 h in control and NPC-treated cultures. Blue = DAPI, green = GFP, red = Aβ, scale = 50 µM.

Given the effect of newborn NPCs on Aβ removal, we compared the effect of NPCs derived from 7-month-old wild type versus 5xFAD mice on Aβ removal by microglia. Microglia were isolated from 7-month-old 5xFAD mice and loaded with Aβ as performed above. Freshly isolated GFP-nestin NPCs were added to the culture for 48 h and Aβ content was quantified as described above. After 48 h, the fraction of microglia with high Aβ content was lower in all experimental conditions, as compared to baseline (Figure 8, ANOVA ** *p* = 0.002). Post hoc analysis revealed a significant effect of both wild type (** *p* = 0.0025) and 5xFAD NPCs (* *p* = 0.012) on Aβ removal. WT NPCs exhibited a non-significant stronger effect in reducing the fraction of microglia with high Aβ content as compared to 5XFAD NPCs. Thus, adult brain NPCs accelerated Aβ removal by microglia.

## 4. Discussion

Resident brain NPCs are considered important players in protecting the brain environment and maintaining homeostasis [36,42]. Within this capacity, they may affect glial cell functions in their microenvironment. Here, we examined NPC–microglial interactions in protecting the brain from amyloid accumulation, as a primary process that initiates AD pathogenesis. We first demonstrated the existence of a subpopulation of microglia with high Aβ phagocytic activity. We showed that newborn WT mouse-derived NPCs induce an increase in Aβ uptake by microglia. Importantly, NPCs’ effect was not distributed evenly among microglia. Rather than affecting the general degree of Aβ uptake by all microglia, we found that NPCs induce microglia to transform into the phenotype characterized by high Aβ phagocytic activity. The supportive effect of NPCs resulted in an increase in the fraction of this microglial population. Finally, we showed that NPCs isolated from 7-month-old wild type mice exhibit similar supportive effects on microglial phagocytosis, whereas NPCs from age-matched 5xFAD mice display functional failure.

Here, we used microglia from 7-month-old 5xFAD brains, a time point of heavy amyloid burden and AD pathology, but prior to neurodegeneration. While the 5xFAD model has obvious limitations, especially the lack of deposition of other misfolded proteins, such as the tau protein and TDP43, which promote neurodegeneration, this model, and the choice of mouse age in our experiments, may represent a relatively early stage of the human disease, with amyloid accumulation and gliosis. 

The AD brain environment activates microglia [43]. This has been demonstrated in multiple ways, including the increased phagocytic activity of latex beads. However, this activation is associated with a decline in microglia functions that are related to AD pathology, and in particular their ability to remove amyloid β [44]. In order to continue the influence of the inflammatory environment after isolating brain microglia for ex vivo experiments, we used LPS. In agreement with the above, LPS caused some increase in latex beads uptake, but reduced significantly Aβ uptake by microglia. This possible discrepancy may be explained by the involvement of different phagocytic pathways, where Aβ uptake occurs mainly via receptor-mediated phagocytosis, whereas latex beads’ uptake is not receptor-mediated [45]. In agreement with this notion, we show here that the trophic effect of NPCs, supporting the phagocytic activity of AD brain-derived microglia, affects Aβ uptake but not latex beads’ uptake.

The notion of the existence of microglial subpopulations with different specializations has been suggested in several studies. Distinct microglial subpopulations have been described in development and adulthood, and with increased heterogeneity in disease states [46,47]. Interestingly, specialized subpopulations characterize states of neurodegeneration and repair [48,49], and a specific subpopulation responds to inflammatory cues [50]. Here, we identified a subpopulation of microglia in the AD brain with high Aβ phagocytic activity. It will be interesting to further characterize this microglial subpopulation, and its role in the pathogenesis of AD. Specifically, while Aβ phagocytosis by human microglia has been demonstrated in multiple studies [51], it will be important to study whether the human AD brain also contains a subpopulation of microglia with high Aβ phagocytic activity and an association with disease-associated microglia (DAM) that were described in AD [48]. 

Indeed, we show here the accumulation of microglia around plaques, and an increased expression of TREM2, as has been shown for DAM. Since reducing the amyloid burden is thought to delay the pathogenesis of AD, at least in the early pathological stages of disease, we hypothesized that this subpopulation may represent a novel therapeutic target for disease modification. Indeed, it has been shown that microglial dysfunction, in their capacity to uptake Aβ in the AD brain, is reversible [52]. It will be important to determine whether increasing the fraction of microglia with high Aβ phagocytic and high Aβ degradation activity may slow down the pathogenesis of AD.

In our study, we found a selective effect of NPCs on microglia with high Aβ phagocytic activity. We showed that NPCs do not increase Aβ phagocytosis in all microglia, but rather increase the fraction of highly phagocytic cells. In agreement, we showed that NPCs induce an increase in the expression of several genes associated with receptor-mediated phagocytosis [53]. However, the full mechanism by which NPCs induce the transformation of microglia with low Aβ phagocytic activity into highly phagocytic cells is not known. Here, we examined the expression of several markers associated with phagocytic activity, but it is not known whether these markers are the mediators of NPCs’ effects. Of note, modulating the TREM2 expression mediates the transformation of microglia into the DAM phenotype [48]. In any case, the selective effect of NPCs on this microglial subpopulation highlights their possible roles in controlling AD brain pathology and as a possible therapeutic target.

While multiple studies have shown the therapeutic properties of NPCs derived from the developing brain, very little is known about the function of resident NPCs in the adult and aging brain. To test that, it is important to study freshly isolated NPCs rather than using cells that have been propagated in vitro [19]. We showed that adult-brain-derived wild type NPCs possess similar trophic properties in supporting microglial Aβ phagocytic activity and in supporting Aβ degradation, as newborn-brain-derived NPCs. It is important to note that NPCs aid these functions in microglia with high Aβ phagocytic activity. This supports the notion that resident NPCs in the adult brain have an important role in maintaining homeostasis by using their therapeutic properties [9,10]. However, NPCs derived from brains inflicted with heavy AD pathology (e.g., 7-month-old 5xFAD mice) exhibited functional failure. This was shown by their inability to support Aβ phagocytosis by microglia and a trend (although not statistically significant) towards a reduced effect on Aβ degradation. These findings possibly have important implications for the pathogenesis of AD. We suggest that the functional failure of NPCs may result in an increased rate of Aβ accumulation in the brain, and a consequent acceleration of disease pathology and the neurodegenerative process.

The failure of NPCs in the 5xFAD mouse brain is not inherent. We have previously shown that NPC failure is acquired in the 5xFAD brain with age, and is reversible upon the propagation of NPCs ex vivo, in conditions that are free from those of the AD brain environment [19]. Therefore, manipulating resident NPCs in the AD brain to overcome their failure in supporting Aβ clearance by microglia may represent an additional potential therapeutic target in AD. Since we examined, here, a specific microglial function related to AD pathogenesis, it is vital to study the trophic and neuroprotective effects of functional NPCs on additional pathogenic processes relevant to AD. Such studies may further underscore the pivotal role of resident NPCs in protecting the adult brain from AD. This notion is in agreement with the observation of a very long time gap between the development of AD brain pathology and the onset of frank neurodegeneration [1,2]. This large gap suggests that the brain may possess means of protective from the injurious consequences of AD pathology.

## 5. Conclusions

In view of our results, we hypothesize that resident NPCs may play an important role in providing protection from Aβ and AD pathology, and that the eventual failure of resident NPCs function may accelerate brain injury and neurodegeneration. Thus, we propose that manipulating NPCs to reverse their acquired failure during the therapeutic time window between Aβ accumulation and neurodegeneration may postpone AD pathogenesis.

## References

[1] Musiek E.S., Holtzman D.M. (2015). Three dimensions of the amyloid hypothesis: Time, space and ‘wingmen’. Nat. Neurosci..

[2] Fleisher A.S., Chen K., Quiroz Y.T., Jakimovich L.J., Gomez M.G., Langois C.M., Langbaum J.B., Ayutyanont N., Roontiva A., Thiyyagura P. (2012). Florbetapir PET analysis of amyloid-beta deposition in the presenilin 1 E280A autosomal dominant Alzheimer’s disease kindred: A cross-sectional study. Lancet Neurol..

[3] Kinney J.W., Bemiller S.M., Murtishaw A.S., Leisgang A.M., Salazar A.M., Lamb B.T. (2018). Inflammation as a central mechanism in Alzheimer’s disease. Alzheimer’s Dement..

[4] Bartels T., De Schepper S., Hong S. (2020). Microglia modulate neurodegeneration in Alzheimer’s and Parkinson’s diseases. Science.

[5] Qin Q., Teng Z., Liu C., Li Q., Yin Y., Tang Y. (2021). TREM2, microglia, and Alzheimer’s disease. Mech. Ageing Dev..

[6] Lee C.Y., Landreth G.E. (2010). The role of microglia in amyloid clearance from the AD brain. J. Neural Transm..

[7] Cai Z., Hussain M.D., Yan L.J. (2014). Microglia, neuroinflammation, and beta-amyloid protein in Alzheimer’s disease. Int. J. Neurosci..

[8] Streit W.J., Khoshbouei H., Bechmann I. (2020). Dystrophic microglia in late-onset Alzheimer’s disease. Glia.

[9] Einstein O., Ben-Hur T. (2008). The changing face of neural stem cell therapy in neurologic diseases. Arch. Neurol..

[10] Ottoboni L., De Feo D., Merlini A., Martino G. (2015). Commonalities in immune modulation between mesenchymal stem cells (MSCs) and neural stem/precursor cells (NPCs). Immunol. Lett..

[11] De Feo D., Merlini A., Laterza C., Martino G. (2012). Neural stem cell transplantation in central nervous system disorders: From cell replacement to neuroprotection. Curr. Opin. Neurol..

[12] Ben-Hur T., Fainstein N., Nishri Y. (2013). Cell-based reparative therapies for multiple sclerosis. Curr. Neurol. Neurosci. Rep..

[13] Ryu J.K., Cho T., Wang Y.T., McLarnon J.G. (2009). Neural progenitor cells attenuate inflammatory reactivity and neuronal loss in an animal model of inflamed AD brain. J. Neuroinflamm..

[14] Zhang Q., Wu H.H., Wang Y., Gu G.J., Zhang W., Xia R. (2016). Neural stem cell transplantation decreases neuroinflammation in a transgenic mouse model of Alzheimer’s disease. J. Neurochem..

[15] McGinley L.M., Kashlan O.N., Bruno E.S., Chen K.S., Hayes J.M., Kashlan S.R., Raykin J., Johe K., Murphy G.G., Feldman E.L. (2018). Human neural stem cell transplantation improves cognition in a murine model of Alzheimer’s disease. Sci. Rep..

[16] Zhang W., Gu G.J., Shen X., Zhang Q., Wang G.M., Wang P.J. (2015). Neural stem cell transplantation enhances mitochondrial biogenesis in a transgenic mouse model of Alzheimer’s disease-like pathology. Neurobiol. Aging.

[17] Kim J.A., Ha S., Shin K.Y., Kim S., Lee K.J., Chong Y.H., Chang K.A., Suh Y.H. (2015). Neural stem cell transplantation at critical period improves learning and memory through restoring synaptic impairment in Alzheimer’s disease mouse model. Cell Death Dis..

[18] Blurton-Jones M., Kitazawa M., Martinez-Coria H., Castello N.A., Muller F.J., Loring J.F., Yamasaki T.R., Poon W.W., Green K.N., LaFerla F.M. (2009). Neural stem cells improve cognition via BDNF in a transgenic model of Alzheimer disease. Proc. Natl. Acad. Sci. USA.

[19] Fainstein N., Dan-Goor N., Ben-Hur T. (2018). Resident brain neural precursor cells develop age-dependent loss of therapeutic functions in Alzheimer’s mice. Neurobiol. Aging.

[20] Mosher K.I., Andres R.H., Fukuhara T., Bieri G., Hasegawa-Moriyama M., He Y., Guzman R., Wyss-Coray T. (2012). Neural progenitor cells regulate microglia functions and activity. Nat. Neurosci..

[21] De Feo D., Merlini A., Brambilla E., Ottoboni L., Laterza C., Menon R., Srinivasan S., Farina C., Garcia Manteiga J.M., Butti E. (2017). Neural precursor cell-secreted TGF-beta2 redirects inflammatory monocyte-derived cells in CNS autoimmunity. J. Clin. Investig..

[22] Peruzzotti-Jametti L., Bernstock J.D., Vicario N., Costa A.S.H., Kwok C.K., Leonardi T., Booty L.M., Bicci I., Balzarotti B., Volpe G. (2018). Macrophage-Derived Extracellular Succinate Licenses Neural Stem Cells to Suppress Chronic Neuroinflammation. Cell Stem Cell.

[23] Bian B., Zhao C., He X., Gong Y., Ren C., Ge L., Zeng Y., Li Q., Chen M., Weng C. (2020). Exosomes derived from neural progenitor cells preserve photoreceptors during retinal degeneration by inactivating microglia. J. Extracell. Vesicles.

[24] Talaveron R., Matarredona E.R., de la Cruz R.R., Macias D., Galvez V., Pastor A.M. (2014). Implanted neural progenitor cells regulate glial reaction to brain injury and establish gap junctions with host glial cells. Glia.

[25] Einstein O., Ben-Menachem-Tzidon O., Mizrachi-Kol R., Reinhartz E., Grigoriadis N., Ben-Hur T. (2006). Survival of neural precursor cells in growth factor-poor environment: Implications for transplantation in chronic disease. Glia.

[26] Goldfarb S., Fainstein N., Ben-Hur T. (2020). Electroconvulsive stimulation attenuates chronic neuroinflammation. JCI Insight.

[27] Goshen I., Kreisel T., Ounallah-Saad H., Renbaum P., Zalzstein Y., Ben-Hur T., Levy-Lahad E., Yirmiya R. (2007). A dual role for interleukin-1 in hippocampal-dependent memory processes. Psychoneuroendocrinology.

[28] Oakley H., Cole S.L., Logan S., Maus E., Shao P., Craft J., Guillozet-Bongaarts A., Ohno M., Disterhoft J., Van Eldik L. (2006). Intraneuronal beta-amyloid aggregates, neurodegeneration, and neuron loss in transgenic mice with five familial Alzheimer’s disease mutations: Potential factors in amyloid plaque formation. J. Neurosci. Off. J. Soc. Neurosci..

[29] Eimer W.A., Vassar R. (2013). Neuron loss in the 5XFAD mouse model of Alzheimer’s disease correlates with intraneuronal Abeta42 accumulation and Caspase-3 activation. Mol. Neurodegener..

[30] Stalder M., Phinney A., Probst A., Sommer B., Staufenbiel M., Jucker M. (1999). Association of microglia with amyloid plaques in brains of APP23 transgenic mice. Am. J. Pathol..

[31] Mawuenyega K.G., Sigurdson W., Ovod V., Munsell L., Kasten T., Morris J.C., Yarasheski K.E., Bateman R.J. (2010). Decreased clearance of CNS beta-amyloid in Alzheimer’s disease. Science.

[32] Brown G.C. (2019). The endotoxin hypothesis of neurodegeneration. J. Neuroinflamm..

[33] Lee J.W., Lee Y.K., Yuk D.Y., Choi D.Y., Ban S.B., Oh K.W., Hong J.T. (2008). Neuro-inflammation induced by lipopolysaccharide causes cognitive impairment through enhancement of beta-amyloid generation. J. Neuroinflamm..

[34] Zhang R., Miller R.G., Gascon R., Champion S., Katz J., Lancero M., Narvaez A., Honrada R., Ruvalcaba D., McGrath M.S. (2009). Circulating endotoxin and systemic immune activation in sporadic amyotrophic lateral sclerosis (sALS). J. Neuroimmunol..

[35] Zhao Y., Jaber V., Lukiw W.J. (2017). Secretory Products of the Human GI Tract Microbiome and Their Potential Impact on Alzheimer’s Disease (AD): Detection of Lipopolysaccharide (LPS) in AD Hippocampus. Front. Cell. Infect. Microbiol..

[36] Zhan X., Stamova B., Jin L.W., De Carli C., Phinney B., Sharp F.R. (2016). Gram-negative bacterial molecules associate with Alzheimer disease pathology. Neurology.

[37] Pluchino S., Martino G. (2008). The therapeutic plasticity of neural stem/precursor cells in multiple sclerosis. J. Neurol. Sci..

[38] Liu X.Y., Yang L.P., Zhao L. (2020). Stem cell therapy for Alzheimer’s disease. World J. Stem Cells.

[39] Liu J., Hjorth E., Zhu M., Calzarossa C., Samuelsson E.B., Schultzberg M., Akesson E. (2013). Interplay between human microglia and neural stem/progenitor cells in an allogeneic co-culture model. J. Cell. Mol. Med..

[40] Mittal K., Eremenko E., Berner O., Elyahu Y., Strominger I., Apelblat D., Nemirovsky A., Spiegel I., Monsonego A. (2019). CD4 T Cells Induce A Subset of MHCII-Expressing Microglia that Attenuates Alzheimer Pathology. iScience.

[41] Mignone J.L., Kukekov V., Chiang A.S., Steindler D., Enikolopov G. (2004). Neural stem and progenitor cells in nestin-GFP transgenic mice. J. Comp. Neurol..

[42] Carletti B., Piemonte F., Rossi F. (2011). Neuroprotection: The emerging concept of restorative neural stem cell biology for the treatment of neurodegenerative diseases. Curr. Neuropharmacol..

[43] Hemonnot A.L., Hua J., Ulmann L., Hirbec H. (2019). Microglia in Alzheimer Disease: Well-Known Targets and New Opportunities. Front. Aging Neurosci..

[44] Fu R., Shen Q., Xu P., Luo J.J., Tang Y. (2014). Phagocytosis of microglia in the central nervous system diseases. Mol. Neurobiol..

[45] Sole-Domenech S., Cruz D.L., Capetillo-Zarate E., Maxfield F.R. (2016). The endocytic pathway in microglia during health, aging and Alzheimer’s disease. Ageing Res. Rev..

[46] Prinz M., Jung S., Priller J. (2019). Microglia Biology: One Century of Evolving Concepts. Cell.

[47] Hammond T.R., Dufort C., Dissing-Olesen L., Giera S., Young A., Wysoker A., Walker A.J., Gergits F., Segel M., Nemesh J. (2019). Single-Cell RNA Sequencing of Microglia throughout the Mouse Lifespan and in the Injured Brain Reveals Complex Cell-State Changes. Immunity.

[48] Keren-Shaul H., Spinrad A., Weiner A., Matcovitch-Natan O., Dvir-Szternfeld R., Ulland T.K., David E., Baruch K., Lara-Astaiso D., Toth B. (2017). A Unique Microglia Type Associated with Restricting Development of Alzheimer’s Disease. Cell.

[49] Olah M., Amor S., Brouwer N., Vinet J., Eggen B., Biber K., Boddeke H.W. (2012). Identification of a microglia phenotype supportive of remyelination. Glia.

[50] Pannell M., Szulzewsky F., Matyash V., Wolf S.A., Kettenmann H. (2014). The subpopulation of microglia sensitive to neurotransmitters/neurohormones is modulated by stimulation with LPS, interferon-gamma, and IL-4. Glia.

[51] Paresce D.M., Ghosh R.N., Maxfield F.R. (1996). Microglial cells internalize aggregates of the Alzheimer’s disease amyloid beta-protein via a scavenger receptor. Neuron.

[52] Daria A., Colombo A., Llovera G., Hampel H., Willem M., Liesz A., Haass C., Tahirovic S. (2017). Young microglia restore amyloid plaque clearance of aged microglia. EMBO J..

[53] Uribe-Querol E., Rosales C. (2020). Phagocytosis: Our Current Understanding of a Universal Biological Process. Front. Immunol..

